# The Renin–Angiotensin System (RAS) in COVID-19 Disease: Where We Are 3 Years after the Beginning of the Pandemic

**DOI:** 10.3390/microorganisms12030583

**Published:** 2024-03-14

**Authors:** Marco Prato, Natalia Tiberti, Cristina Mazzi, Federico Gobbi, Chiara Piubelli, Silvia Stefania Longoni

**Affiliations:** 1 Department of Infectious, Tropical Diseases and Microbiology, IRCCS Sacro Cuore Don Calabria Hospital, Negrar di Valpolicella, 37024 Verona, Italy; 2 Centre for Clinical Research, IRCCS Sacro Cuore Don Calabria Hospital, Negrar di Valpolicella, 37024 Verona, Italy

**Keywords:** COVID-19, RAS pathway, ACE, ACE2, AngII, Ang1-7

## Abstract

The RAS is a hormonal system playing a pivotal role in the control of blood pressure and electrolyte homeostasis, the alteration of which is associated with different pathologies, including acute respiratory distress syndrome (ARDS). As such, it is not surprising that a number of studies have attempted to elucidate the role and balance of the renin–angiotensin system (RAS) in COVID-19. In this review article, we will describe the evidence collected regarding the two main enzymes of the RAS (i.e., ACE and ACE2) and their principal molecular products (i.e., AngII and Ang1-7) in SARS-CoV-2 infection, with the overarching goal of drawing conclusions on their possible role as clinical markers in association with disease severity, progression, and outcome. Moreover, we will bring into the picture new experimental data regarding the systemic activity of ACE and ACE2 as well as the concentration of AngII and Ang1-7 in a cohort of 47 COVID-19 patients hospitalized at the IRCCS Sacro Cuore-Don Calabria Hospital (Negrar, Italy) between March and April 2020. Finally, we will discuss the possibility of considering this systemic pathway as a clinical marker for COVID-19.

## 1. The Renin–Angiotensin System

Since the discovery of renin at the end of the 19th century, the renin–angiotensin system (RAS) has been largely studied for its important role in the regulation of blood pressure. The RAS is a hormonal system strictly involved in the control of blood pressure and electrolyte homeostasis [1]. Since it is present in several cells and tissues, the dysregulation of the RAS has been hypothesized to be involved in a number of different pathologies affecting multiple organs such as the liver, pancreas, skeletal muscle, kidney, lungs, blood vessels, heart, bone marrow, and nervous system [2,3]. 

**Figure 1:**
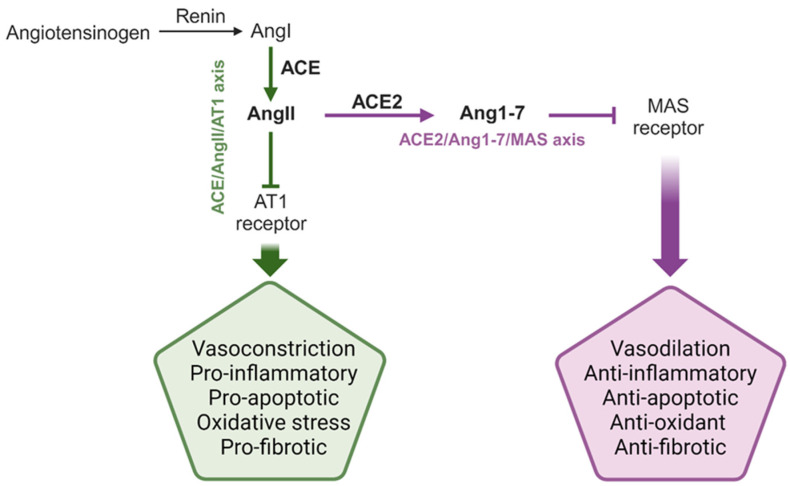
Schematic representation of the classical and alternative RAS. The main enzymes, hormones, and peptide products of the system are reported, together with the main systemic and local effects mediated by the RAS. The two enzymes and the two products described in this review manuscript are highlighted in bold. AngI: angiotensin I; AngII: angiotensin II; Ang1-7: angiotensin 1-7; ACE: angiotensin converting enzyme; ACE2: angiotensin converting enzyme 2. This figure was created using Biorender.com.

The pathway comprises (i) a classic RAS, leading to the production of angiotensin II (AngII) through the ACE/AngII/AT1 axis; (ii) an alternative RAS, involving the production of angiotensin 1-7 (Ang1-7) through the ACE2/Ang1-7/MAS axis (Figure 1). 

Renin and angiotensin-converting enzyme (ACE) are the two key enzymes of the classic RAS. The first is produced by the kidney and hydrolases the pro-hormone angiotensinogen, released by the liver, into angiotensin I (AngI) [3], while the second enzyme catalyzes the cleavage of AngII from AngI [3] (Figure 1). Depending on the target organ, AngII binding to its transmembrane AT1 receptor induces vasoconstriction (in vascular smooth muscles), sodium reabsorption (in the kidney), or aldosterone production (in the adrenal cortex) [3,4]. Indeed, AngII is a strong vasoconstrictor and pro-inflammatory hormone that acts both at renal and vascular levels to enhance the peripheral resistance of vessels, ultimately leading to high blood pressure (HBP) [5]. At the local level (i.e., heart, brain, and liver), it mediates local pathological events associated with inflammation, reactive oxygen species (ROS) production, and fibrosis, for instance [1].

The alternative RAS was first described in the early 2000s following the discovery of angiotensin-converting enzyme 2 (ACE2) as an ACE counterpart. Indeed, ACE2 is responsible for the hydrolysis of AngII into Ang1-7 [6,7,8,9], which counteracts AngII effects by mediating anti-proliferative, anti-inflammatory, and anti-apoptotic responses [9,10], through binding to its natural transmembrane receptor MAS [2,3,11] (Figure 1). 

Based on these premises, it is not surprising that the systemic or local activity of ACE and ACE2 and the concentration of AngII and Ang1-7 have been described in many human tissues and organs, and over the years, their involvement in cardiovascular diseases (CVD), hypertension, type 2 diabetes, and chronic kidney disease has been gradually elucidated [12,13,14,15]. Consequently, ACE inhibitors (e.g., Captopril) and angiotensin II type 1 (AT1) receptor blockers (ARBs) (e.g., Losartan) represent, nowadays, important therapeutic strategies against CVD, chronic kidney disease, and type 2 diabetes [16]. The RAS has also been proposed as an important player in the pathogenesis of acute respiratory distress syndrome (ARDS), the most severe form of acute lung injury [12,17]. In this particular context, it has been proposed that ACE2 activity could be necessary to counteract AngII, which is initially essential for an effective defense, but when chronically over-expressed could compromise the host immune defense, leading to potentially overwhelming bacterial infection [18]. Pathogens are among the major risk factors for ARDS, since pneumonia and non-pulmonary sepsis are, overall, responsible for 65–80% of all ARDS cases [19]. It might, thus, not be surprising that ACE2, which is highly expressed by oral cavity and lung epithelial cells [20], represents a molecular target for many respiratory pathogens. Consequently, the RAS, and particularly ACE2, have also been investigated in respiratory viral infections, including those caused by coronaviruses. 

## 2. The RAS and Viral Respiratory Infections

Viral infections play an important role in ARDS onset, probably through the modulation and imbalance of the RAS [21]. It is known that the ACE2/Ang1-7/MAS axis is of particular importance in maintaining an equilibrium between an effective immune defense against microbial infections and in preventing immune-mediated tissue damage [17,18]. Different studies have attempted to elucidate the role of the RAS in lung pathologies associated with different viral infections in order to propose biomarkers of clinical utility. Indeed, an RAS imbalance has been described in patients with avian influenza (H5N1, H7N9), influenza A (H1N1), and hand, foot, and mouth disease (HFMD) caused by Coxsackie virus A16 (CA16) and enterovirus 71 (EV71). Significantly higher systemic concentrations of AngII have been reported in patients infected with H5N1 [22], H7N1, and H1N1 [23] when compared with uninfected control subjects. Interestingly, H7N1, which causes acute respiratory failures in humans, triggered higher and sustained circulating AngII levels than H1N1, while in both cases, plasma AngII concentrations were positively correlated with viral load [23]. These observations were also confirmed using a murine model since, in mice, H5N1 infection triggered a reduction in ACE2 expression in the lungs and an increase in AngII concentration in serum. During H7N1 and H5N1 infections, raised systemic AngII levels were associated with longer hospitalization and increased severity and mortality in both patients and mice [22,23]. These observations were corroborated by additional experimental data obtained using mice knock-out for ACE2 (ACE2-ko). Indeed, in agreement with increased circulating (c)AngII, ACE2-ko mice challenged with H5N1 presented a higher mortality rate and a more severe lung pathology, which was attenuated following treatment with recombinant ACE2 (rACE2) [22], suggesting a beneficial role of this enzyme in alleviating lung injuries during respiratory viral infections [22]. 

Serum concentrations of AngII were also found to be higher in pediatric patients with HFMD than in healthy controls, particularly in those with a severe clinical presentation [24], while local AngII (i.e., brain, skeletal muscle, and lungs) increased with disease progression in EV71-infected mice [24]. 

The systemic RAS balance was also investigated in severe acute respiratory syndrome (SARS) caused by a coronavirus (SARS-CoV) that appeared for the first time in 2002, causing potentially fatal lung injury and ARDS [25]. Interestingly, mACE2 (membrane ACE2) was identified as the virus receptor on many different types of host cells [25,26,27], mediating viral entry through the binding of the spike (S) protein of the viral envelope [26,28,29,30]. The same mechanism was also described for the human coronavirus NL63 (HCoV-NL63) discovered in the Netherlands in 2004 [31], even though it binds less efficiently to ACE2 [30].

Different studies have shown a reduction in ACE2 expression in the lungs following SARS-CoV infection in mice [30,32] and an association between this local ACE2 reduction and the development of severe acute respiratory failure [33]. This phenomenon was shown to be associated with the binding of the spike protein to ACE2 and with a subsequent shedding of ACE2 from the cell surface [30,32,34], suggesting that SARS-CoV could downregulate its own receptor. 

A functional role for SARS-CoV S protein in worsening lung pathology, likely by deregulating the RAS, was also proposed [32] since mice treated with the Spike–Fc protein presented increased AngII in the lungs and worsened lung function, while blocking the RAS using a specific AT1 receptor inhibitor attenuated the development of acute lung failure [32]. This agrees with ACE2-ko mice presenting reduced pathologic alterations in the lungs following the injection of the Spike–Fc protein. Moreover, the treatment with rACE2 attenuated lung injury in both ACE2-ko and wild-type mice, suggesting the possibility of using rACE2 to modulate the RAS also during SARS-CoV infection [33].

Unfortunately, mainly as a consequence of SARS-CoV’s high mortality rate and the relatively small number of registered cases, a more in-depth evaluation of RAS balance at the systemic level in clinical samples and the prognostic value of circulating RAS molecules in SARS-CoV patients has not been addressed.

### 2.1. RAS and COVID-19

Based on these observations, it is not surprising that when the entire world was exposed to the outbreak of a new coronavirus disease (COVID-19), caused by the previously unknown human coronavirus SARS-CoV-2, the attention of researchers was also drawn to the role and balance of the RAS during this novel viral infection [35,36,37,38,39,40]. 

Similarly to other coronaviruses, SARS-CoV-2 uses ACE2 expressed on host cells as the main receptor for cell invasion through the binding of the S protein of the viral envelope [41]. The cell surface expression of ACE2 is higher in alveolar pneumocytes, although a broad spectrum of human cells, including enterocytes and vascular endothelial cells, can express this receptor, offering multiple targets for viral entrance [42]. This wide distribution of ACE2 across cell types could, at least partly, explain the broad range of clinical manifestations of COVID-19, spanning from respiratory to gastrointestinal manifestations [35]. As already observed for SARS-CoV, SARS-CoV-2 binding to its receptor results in ACE2 downregulation at the transcriptional and protein expression level in vitro [43,44]. It was then hypothesized that this ACE2 downregulation could potentially lead to increased ACE, which, in turn, could promote the cytokine storm observed in patients with lung injury [36,45], although direct evidence is still missing. 

Since the beginning of the pandemic, many authors have speculated about the modulation of the RAS by SARS-CoV-2 based on the following: (i) the important role of the RAS in the pathological mechanisms of acute lung injury and ARDS; (ii) the key role of ACE2 receptor in viral entrance; and (iii) the proposed ability of rACE2 to reduce lung injury in SARS-CoV infection. It has also been proposed that alterations in the RAS could predict the outcome of the disease [35,36,37,38,39], which is based on the hypothesis that, at the serum level, there would have been the same RAS imbalance [46,47] observed at the tissue level [48]. 

Many studies have tried to elucidate this putative link between systemic RAS imbalance and COVID-19 infection, with the main aim of finding prognostic markers of clinical utility as well as improving treatment strategies. Despite the huge efforts made by the scientific community, some limitations concerning the investigation of the RAS in COVID-19 arose, including the lack of a standardized strategy to determine circulating enzymatic activity and peptide concentration, and the lack of the systematic measurement of multiple RAS molecules. Moreover, most studies have been limited by a small sample size, by the lack of an in-depth clinical and demographic characterization of the study cohort, and by high heterogeneity in the timing of blood collection. Consequently, a straightforward comparison of the results obtained in the different studies can be found difficult. 

In the following paragraphs, we will try to summarize the principal studies investigating circulating ACE, ACE2, AngII, and Ang1-7 in clinical samples from COVID-19 patients in order to highlight reproducible pieces of evidence across studies as well as potential inconsistencies that need to be further explored. Particular attention will be paid to patients’ stratification based on disease severity and outcome to comprehensively evaluate the association of this metabolic pathway with the course of the disease.

#### 2.1.1. Alterations in Circulating RAS Molecules in COVID-19 Patients Compared to Healthy Subjects 

In the effort to evaluate a potential systemic imbalance in the RAS associated with COVID-19, a number of studies have measured and compared circulating RAS molecules between patients and control subjects (Table 1). 

In an early report dating back to February 2020, Liu and co-workers reported increased levels of plasmatic AngII and decreased Ang1-7 in twelve SARS-CoV-2 patients presenting pneumonia compared with healthy subjects [49]. In agreement, raised AngII levels were also observed in critically ill patients with COVID-19 compared to critically ill patients without SARS-CoV-2 infection [63]. Similar results were obtained in an independent cohort of hospitalized patients [50], in which raised AngII concentrations were accompanied by decreased Ang1-7 and ACE2 concentrations but increased ACE2 activity in patients compared to controls [50].

Partly contrasting results were instead reported in severely ill hospitalized COVID-19 patients, who presented reduced cACE2 activity in both plasma and saliva when compared to negative controls [51]. In agreement with this reduced cACE2 activity, COVID-19 patients also displayed decreased Ang1-7 concentrations, indicating an overall downregulation of the alternative RAS pathway in this population. Interestingly, this reduction was accompanied by significantly increased concentrations of cACE2, suggesting that the reduction in ACE2 activity was not due to a reduced enzyme concentration. Moreover, treatment with convalescent plasma was reported to restore ACE2 activity after 60 days [51]. The systemic alteration in ACE2 concentration was not mirrored at the local level since no differences in ACE2 quantification in lung tissue from patients who died from COVID-19 or other pathological conditions were detected [51]. Unfortunately, disagreement among published data was also observed regarding cACE2 concentration, with two studies reporting lower [50,52] and four reporting higher cACE2 concentrations [51,53,54,55] in COVID-19 patients compared to healthy controls (Table 1). It might be worth reminding readers that enzyme concentration does not reflect activity. While the concentration of cACE2 could be of interest for speculation about the ability of the human body to capture the virus and avoid cell infection, it might only be of limited utility for the evaluation of the RAS balance since a high concentration of cACE2 or cACE does not directly reflect a high synthesis of their products in serum.

An overall potential downregulation of the RAS was reported by Henry and colleagues [65], who observed decreased AngI and Ang1-7 in COVID-19 patients upon hospital admission compared to healthy controls, even though the enzymatic activity and concentration of ACE were not significantly different, and the activity and concentration of ACE2 were not determined [65]. Avanoglu Guler et al. did not find any significant differences in ACE activity in infected subjects compared to healthy controls [57,64]. Lower cACE concentrations were instead observed in COVID-19 patients compared with healthy controls [58], even though this was accompanied by significantly high ACE2 and Ang1-7 concentrations in patients [58]. 

Contrasting results have, however, progressively been published. For instance, COVID-19 patients were reported to have the same [56] or even lower blood AngII concentrations than healthy controls [54]. In this latter study, COVID-19 patients, who were critically ill with respiratory failure and admitted to the intensive care unit (ICU), presented increased plasma ACE2 concentrations when compared to healthy controls. This increase was also associated with decreased AngII and, in contrast with what was reported by others [51], increased Ang1-7 formation, as determined by equilibration assay [54]. Lastly, increased activity of cACE has not been associated with COVID-19 [53,66]; rather, it has been shown to be lower in COVID-19 patients when compared to healthy controls [59]. 

#### 2.1.2. Association between RAS Dysregulation and COVID-19 Severity or Outcome

When systemic AngII concentration was evaluated according to the severity of COVID-19, the reported data showed, again, a certain level of disagreement. An important bias in the early studies was the definition of severity itself. Indeed, especially at the beginning of the pandemic and before WHO guidelines were published [67], different criteria were used to classify hospitalized patients as mild, moderate, severe, or, in some cases, critical. It is, thus, not surprising that contrasting, or even opposite, results have been reported in the literature. It is also important to mention that, with respect to disease severity, cACE2 was the most investigated RAS molecule, while only a few studies also took into consideration cACE, AngII, and Ang1-7 activity or systemic concentration. A summary of the main results obtained for the evaluation of the RAS associated with disease severity is reported in Table 2. 

In a small population of 12 patients, plasma AngII was shown to be associated with viral load and lung capacity since a significant negative correlation was reported with both the Ct (cycle threshold) value (Spearman rho = −0.669) and PaO_2_/FiO_2_ (correlation coefficient = −0.545) [49]. Such a relation was confirmed in a larger population encompassing 82 COVID-19 patients [63], in which significantly higher AngII concentrations, but not renin, were observed in severe and critically ill patients compared to those suffering from a mild infection [63,64]. Reindl-Schwaighofer and collaborators also reported higher AngII concentrations in severe COVID-19 patients than in non-severe patients [69]. Similarly, in critically ill COVID-19 patients, AngII levels upon admission were higher than those in severe patients and significantly decreased at discharge [66], while no differences were detected for ACE, ACE2, or Ang1-7 (Table 2). Interesting data are also available regarding cACE2 and COVID-19 severity since Akin and collaborators reported significantly higher cACE2 activity in severe patients than in non-severe ones [68] (Table 2). On the other hand, cACE activity has been reported to be lower in non-severe COVID-19 patients compared to severe patients [60] (Table 2).

A different trend was instead observed in patients suffering from hypertension [72]. In this particular population, severe cases presented higher ACE2 levels compared to both mild and moderate cases and decreased circulating AngII compared to mild subjects [72], suggesting that increased circulating ACE2 could be indicative of a more severe disease in patients under antihypertensive treatment. Indeed, a significant, although only moderate, positive correlation between plasmatic ACE2 and other indices of severity (i.e., D-dimer and length of hospitalization) was reported [72]. It is worth mentioning that different studies have reported that disease severity upon admission showed a stronger association with the presence of comorbidities, i.e., older age, hypertension, and body mass index (BMI), rather than with RAS circulating molecules [57,60,68,70]. Moreover, both ACE2 and AngII were partly influenced by the type of antihypertensive treatment since they were higher in patients under ARBs treatment compared to those taking ACEi [72]. In agreement with this, Files and colleagues did not observe differences between moderate and severe acute hypoxic respiratory failure (AHRF) COVID-19 patients for cACE2 activity and AngII and Ang1-7 concentrations [62]. Patients’ stratification according to COVID-19 severity revealed lower cACE concentrations in mild compared to severe COVID-19 patients (*p* = 0.054), while among COVID-19 patients, those who developed cutaneous symptoms presented higher cACE2 concentrations than the rest of the patients (gastrointestinal and pulmonary) [73]. On the other hand, serum cACE and cACE2 concentrations did not show an association with the outcome of the diseases [53]. 

The association of soluble ACE activity and COVID-19 severity is also controversial since both unaltered and decreased activity were reported in severe patients in different studies [57,60]. Moreover, higher cACE2 activities and AngII/Ang1-7 ratios—a parameter frequently used as a surrogate for cACE2 activity—were observed in severe patients when compared with patients with influenza [69].

Interestingly, the contrasting data reported in the literature about the activity and the concentration of cACE2 could be explained by its time dependence. Reindl-Schwaighofer and collaborators reported lower cACE2 activities in blood taken early during hospitalization (i.e., days 0–3) compared with blood taken later (i.e., days 9–11) from the same COVID-19 patients, both severe and non-severe. Interestingly, when measured upon admission, they did not observe any significant differences in cACE2 activity between severe and non-severe patients, while they reported a 7-fold increase in severe patients during the course of the disease, with ACE2 being increased later during hospitalization [69].

These data suggest a pathophysiological role for cACE2, potentially inflammation-driven, aimed at counterbalancing an excess of AngII during the course of the infection. AngII and Ang1-7, in fact, were significantly higher and lower, respectively, in patients with severe COVID-19 upon admission and reverted a few days after hospitalization. This observation is reflected in the 4.5-fold higher Ang1-7/AngII ratio in severe COVID-19 patients 9–11 days after admission [69]. 

A trend, although not significant, toward increased levels of circulating AngII and decreased Ang1-7 was also observed in association with COVID-19 mortality in a cohort of 74 hospitalized patients suffering from mild or severe COVID-19 [50]. Interestingly, this trend translated into a significantly higher AngII/Ang1-7 ratio in patients who died compared to survivors, which also displayed a moderate potential as a predictor of mortality (AUC of 0.657). Indeed, patients with Ang II/Ang1-7 ≥ 3.45 had a 5-fold increased risk of mortality [50] (Table 3). Importantly, these observations were independent of the use of ACE inhibitors or ARBs [50]. 

Higher cACE2 levels were reported in COVID-19 patients with fatal outcomes compared to survivors [74] and in intubated patients compared with non-intubated COVID-19 patients [70]. Oppositely, Wuang and colleagues observed no significant differences in cACE2 concentrations between COVID-19 patients who survived compared to those who died from COVID-19 [55]. 

### 2.2. Systemic RAS during COVID-19: New Experimental Data

As already pointed out, an important limitation common to most of the published studies is the lack of measurement of the four main RAS players in the same cohort of patients. In our group, we tried to contribute to filling this gap by measuring cACE and cACE2 enzymatic activity, as well as AngII and Ang1-7 concentrations in the serum of SARS-CoV-2 patients (*n* = 47) and healthy controls (*n* = 12). COVID-19 patients were admitted to our hospital during the first COVID-19 wave from March to April 2020. The two groups—COVID-19 patients and controls—were matched for sex, with 55% males in the COVID-19 group and 50% in the control group, but not for age, with median ages of 75 (68–76) and 53.5 (52–57.25) (median and interquartile range), respectively. Additionally, 81% of COVID-19 patients presented comorbidities, while 37% were under ACEi/ARBs treatment. Data concerning the treatment and comorbidities for non-COVID-19 controls were not available. Based on a modified WHO score (WHO Working Group on the Clinical Characterization and Management of COVID-19 infection, 2020), COVID-19 patients were classified as mild (score 4, *n* = 18), moderate (score 5, *n* = 23), or severe (score ≥ 6, *n* = 6), as already reported elsewhere [75]. Patients were also stratified based on the clinical course during hospitalization (i.e., worsened (*n* = 19) or improved (*n* = 28)). The full demographic and clinical characteristics of the studied population as well as the laboratory findings are reported in the Supplementary Dataset and Supplementary S1. All subjects signed written informed consent, and the study was approved by the Ethical Committee of Verona and Rovigo provinces under protocol no. 63471/2020. 

ACE2 activity was measured by an in-house method, while ACE activity was determined using the Angiotensin I Converting Enzyme (ACE) Activity Assay Kit (Fluorimetric) (Sigma-Aldrich, Merck KGaA, Darmstadt, Germany). The concentrations of AngII and Ang1-7 were measured using commercial ELISA kits (MybioSource, Inc., San Diego, CA, USA). A full description of the materials and methods used is reported in Supplementary Material S1.

**Figure 2:**
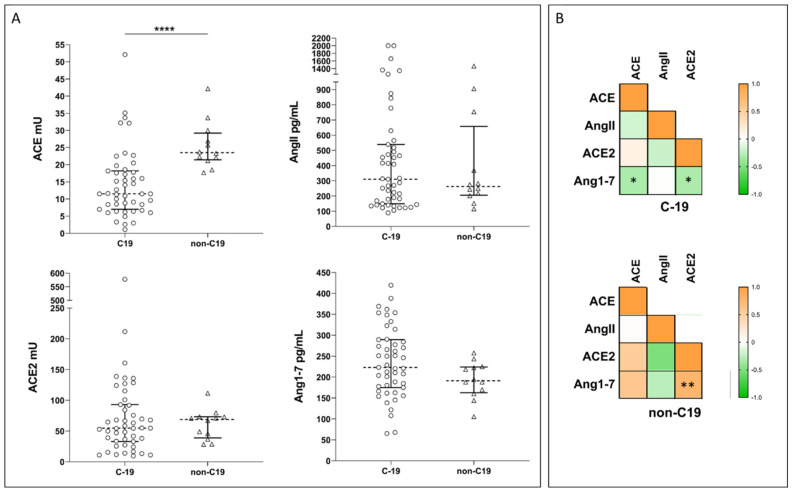
(**A**) Plots showing ACE and ACE2 activities and Ang1-7 and AngII concentrations in COVID-19 patients (C-19) and healthy controls (non-C19). Dotted line represents the median, and error bars represent the interquartile range. Statistical comparisons were computed using the Mann–Whitney test. **** = *p*-value < 0.0001. (**B**) Spearman correlation matrix for ACE and ACE2 activities, and Ang1-7 and AngII concentrations in COVID-19 patients (C-19) and healthy controls (non-C19). Color scale represents Spearman *rho* coefficient. Stars on plot indicate statistically significant correlations. * = *p*-value < 0.05; ** = *p*-value < 0.005.

Upon admission, we observed significantly lower ACE activities in COVID-19 patients compared with non-COVID-19 controls, while ACE2 activities and AngII and Ang1-7 concentrations did not differ between the two groups (Figure 2A). 

In our population, the four molecules displayed different correlation profiles in COVID-19 patients and healthy controls. Indeed, in COVID-19, we observed a weak statistically significant negative correlation between Ang1-7 concentration and both cACE and cACE2, while in controls, Ang1-7 concentration was positively correlated with cACE2 (Figure 2B). COVID-19 patients were further stratified based on disease severity (i.e., severe *n* = 6, moderate *n* = 23, or mild *n* = 18), following a modified WHO score (Supplementary Material S1), or according to the clinical course during hospitalization (i.e., worsened *n* = 19 or improved *n* = 28) [75]. Despite these stratifications, we did not observe any significant differences between the subgroups for any of the measured enzymes or products. Similarly, we did not observe any significant difference in the AngII/Ang1-7 ratio.

**Figure 3:**
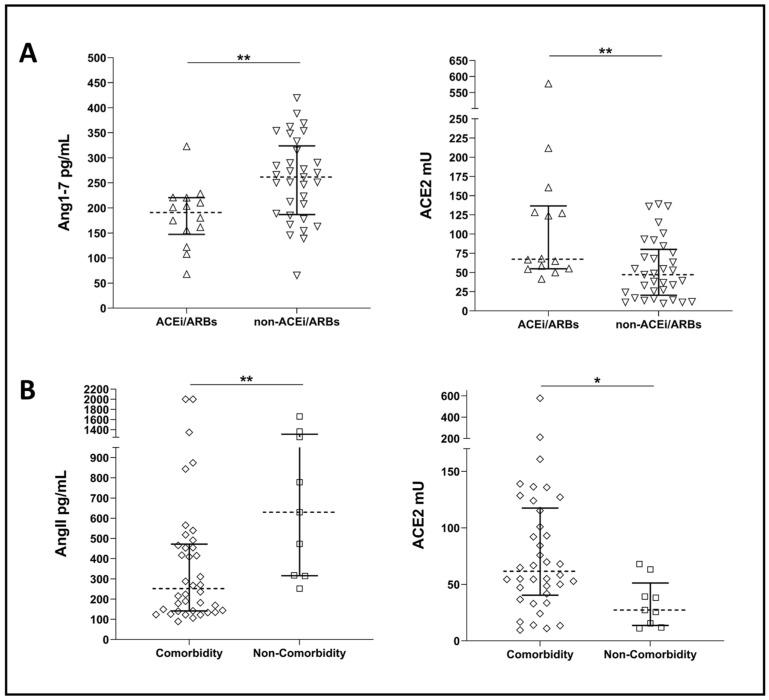
(**A**) Plots showing Ang1-7 concentrations and ACE2 activity in COVID-19 patients under ACEi/ARBs treatment (ACEi/ARBs) and COVID-19 patients not under ACEi/ARBs treatment (non- ACEi/ARBs). (**B**) Plots showing AngII concentrations and ACE2 activities in COVID-19 patients with comorbidities (comorbidity) and COVID-19 patients without comorbidities (non-comorbidity). Dotted line represents median, and error bars represent interquartile range. Statistical comparisons were computed using the Mann–Whitney test. * = *p*-value < 0.05; ** = *p*-value < 0.01.

Our COVID-19 population comprised 30% of subjects under ACE inhibitors (ACEi) or angiotensin receptor blockers (ARBs) treatment. As expected, we observed a significant difference in cACE2 activities and Ang1-7 concentrations in COVID-19 patients under ACEi or ARBs treatment. Specifically, we found higher cACE2 activities and lower Ang1-7 concentrations in those patients under ACEi/ARBs treatment (Figure 3A). 

Our COVID-19 population comprised 38 patients (81%) presenting with comorbidities upon admission, mainly cardiovascular diseases (Table S1). Significantly higher ACE2 activities and lower AngII concentrations were observed in COVID-19 patients with comorbidities compared to those without, even though this last group had a small sample size (i.e., *n* = 9) (Figure 3B).

In an attempt to establish whether some demographic or clinical factors could affect ACE and ACE2 activities or AngII and Agn1-7 concentrations in COVID-19 patients, we performed univariable and multivariable linear regression analyses (Tables S2 and S3). The univariable analysis showed a significant association of ACE2 and its product, Ang1-7, with ACE inhibitors, patients’ age, and comorbidities, although only ACE inhibitors were still significant in the multivariable model. Among the assessed variables, only sex showed a weak association with ACE, while AngII was associated with sex and the presence of co-morbidities (Table S2), the latter was also significant in the multivariable analysis (Table S3). Our data suggest that during COVID-19, the RAS is much more likely to be modulated by the use of ACEi/ARBs or by the presence of co-morbidities (for AngII) than other factors (Table S3). Despite the fact that, in univariable analysis, sex seemed to influence the levels of AngII, we did not observe any significant influence in the multivariable model (Table S3). 

Our study presents some limitations: (i) a low number of analyzed subjects; (ii) COVID-19 patients and control subjects were not matched by age, comorbidities, or treatments; (iii) there was a high prevalence of co-morbidities (80%) among COVID-19 patients; (iv) COVID-19 patients differed in their baseline medical therapy since 30% of them were on ACEi or angiotensin receptor blockers (ARBs); and (v) information about comorbidities or ACEi/ARBs treatment in the control subjects was not available. Despite these limitations, we showed that, overall, the RAS is not modulated during COVID-19 disease, except for the activity of ACE, which is reduced during SARS-CoV2 infection early during hospitalization. 

## 3. Discussion and Concluding Remarks

Based on the current knowledge of the RAS in other diseases, it would be reasonable to think that higher ACE2 activity should lead to higher levels of Ang1-7, and likewise, higher ACE activity should correspond to higher levels of AngII. Moreover, high levels of AngII are known to lead to pro-inflammatory and pro-fibrotic effects, while Ang1-7 is associated with anti-inflammatory and anti-fibrotic effects [3,76,77,78]. Based on this knowledge, we expected COVID-19 to be associated with higher activity of ACE and, consequently, higher AngII levels, lower ACE2 activity, and lower Ang1-7 concentration. Additionally, we also expected this profile to be related to disease severity and outcomes. However, after a careful revision of the literature and some additional experimental data generated in our laboratory, it now seems evident that there is not a clear association between COVID-19 and a specific RAS dysregulation, as indicated by the contrasting results reported across studies (Table 1, Table 2 and Table 3). On the contrary, a number of studies investigating the RAS during either COVID-19 or other pathological conditions seem to suggest that co-morbidities or ACEi/ARBs treatment might exert a strong effect on the RAS balance [79,80,81,82,83]. To understand the role of co-morbidities in the regulation of the RAS, it is worth remembering that ACE2 is shed from the membrane surface and released into the blood stream following tissue damage [35,84]. It could be hypothesized that since SARS-CoV-2 uses ACE2 as a cellular entrance gate, the catalytic activity, at the tissue level, of this enzyme might be altered [85]. This could prevent the massive release of ACE2 into the blood stream, which is, instead, observed in other pathological conditions characterized by tissue damage [85], such as CVD, hypertension, type 2 diabetes, and chronic kidney disease, for which circulating ACE2 serves as a prognostic marker [86,87,88,89].

In fact, it has been reported that CVD patients present higher levels of cACE2 activity than healthy controls and that cACE2 activity correlates with the severity of the disease [90,91]. Most of the studies dealing with COVID-19 often did not fully describe the healthy control groups, especially with regard to the presence of comorbidities or treatment with RAS blockers. As reported in Table 1, a higher cACE2 activity was observed in COVID-19 patients when compared with healthy subjects with no history of CVD or previous treatment with ACEi/ARBs [50,54]. In our experimental data, instead, we did not observe differences in ACE2 activity in COVID-19 patients compared with healthy controls (Figure 2); however, we cannot exclude the presence of co-morbidities, such as CVD, in our control group since the information was not available.

Nonetheless, in our population of COVID-19 patients, we did observe significantly higher ACE2 activity in patients presenting at least one comorbidity compared to those presenting only with COVID-19 (Figure 3). cACE2 concentration was also reported to be higher in COVID-19 patients with pre-existing co-morbidities, such as CVD, hypertension, or kidney diseases, compared with patients without co-morbidities [70] as well as healthy controls with no history of comorbidities or RAS blocker treatment [53]. Opposite to what was believed at the beginning of the pandemic, scientific evidence has demonstrated the lack of association between the use of RAS blockers and susceptibility to SARS-CoV2 infection or disease severity [92,93,94,95]. Indeed, patients presenting CVD or kidney-related co-morbidities, which are often treated with RAS blockers such as ACEi/ARBs, calcium channel blockers, or beta-blockers, do not present less severe COVID-19 clinical presentation. No differences were recorded in cACE2 concentrations when comparing COVID-19 patients treated with ACEi/ARBs to COVID-19 patients without treatment [72].

It could be hypothesized that in order to counterbalance coronavirus’s negative effects on the RAS, the kallikrein–kinin system (KKS) could be activated [85,96]. KKS is a system responsible for the production of bradykinin [85,97], a pro-inflammatory molecule and potent vasodilator [98], which has been found to be increased in COVID-19 patients [85,99]. Unfortunately, only a few studies have investigated the role of KKS and RAS during COVID-19, and further investigations should be performed. 

It could, thus, be speculated that factors other than the SARS-CoV-2 infection might affect circulating ACE, ACE2, AngII, and Ang1-7 levels than COVID-19 itself, as also supported by our data.

This lack of association between a specific RAS imbalance and COVID-19 disease and the high disagreement between the results reported in the literature could be partly explained by a number of reasons. First, at the beginning of the pandemic, it was difficult to properly design and define the study cohorts, particularly for controls, which, most of the time, did not perfectly match COVID-19 patients in terms of age, comorbidities, and/or treatments. Second, until the release of the WHO’s official guidelines in June 2020 [67], each study employed different classification criteria for the definition of severity. Third, the heterogeneity in the procedure for sample collection, such as the type of tubes, the use of protease inhibitors, the time delay between sample collection and analysis, the use of fresh or frozen samples, and the procedures for sample storage, might have influenced the results of the analyses.

In conclusion, based on our observations and a careful analysis of the literature, at the serum level, the RAS pathway does not present a clear imbalance in association with SARS-CoV-2 infection. From the studies reported in this review, it is not possible to exclude an RAS imbalance at the tissue level, which, however, would be of limited utility as a prognostic marker.

Thus, based on the pieces of evidence present in the literature, soluble ACE, ACE2, AngII, or Ang1-7 cannot currently be considered as markers for the diagnosis or prognosis of COVID-19. A number of additional factors (such as age, sex, co-morbidities, and treatments) are indeed likely to influence the RAS system during COVID-19 infection and deserve a more in-depth investigation. Moreover, research could also be extended to the KKS, as the natural counterbalance to the RAS, in order to build a more complete picture of the mechanisms leading to RAS alterations during COVID-19 infection.

## Figures and Tables

**Table 1:** Modulation of RAS molecules in COVID-19 patients during hospitalization compared to controls.

COVID-19	Controls	ACE (Act.)	ACE (Conc.)	ACE2 (Act.)	ACE2 (Conc.)	AngII (Conc.)	Ang1-7 (Conc.)	AngII/Ang1-7 (Ratio)	Ref.
Hospitalized, *n* = 12	Healthy, *n* = 8					↑			[49]
Hospitalized, *n* = 74	Healthy, *n* = 55			↑	↓	↑	↓	↑	[50]
Hospitalized, *n* = 16	Healthy, *n* = 17			↓	↑		↓		[51]
Hospitalized, *n* = 519	Healthy, *n* = 201				↓				[52]
Hospitalized, *n* = 114	Healthy, *n* = 10		≈		↑				[53]
Hospitalized, *n* = 10	Healthy, *n* = 5			↑		↓	↑		[54]
Hospitalized, *n* = 242	Healthy, *n* = 38					↑			[55]
Hospitalized, *n* = 30	Healthy, *n* = 14					≈			[56]
Hospitalized, *n* = 55	Healthy, *n* = 18	≈							[57]
Hospitalized, *n* = 84	Healthy, *n* = 18		↓		↑	≈	↑		[58]
Hospitalized, *n* = 81	Healthy, *n* = 316	↓							[59]
Hospitalized, *n* = 136	Healthy, *n* = 60	↓							[60]
Hospitalized, *n* = 112	Healthy, *n* = 27					↓			[61]
Hospitalized, *n* = 29	Healthy, *n* = 15				↓	↑	≈		[42]
Hospitalized, *n* = 19	ARF non-COVID19, *n* = 19	↑							[62]
Hospitalized, *n* = 82	Critically ill non-COVID19 *n*= 12					↑			[63]
Hospitalized, *n* = 52	Sick non-COVID19 *n*= 27	≈	≈						[64]
Hospitalized, *n* = 27	Sick non-COVID19 *n*= 14						↓		[65]

Act.—activity; Conc.—concentration; Ref.—reference; ↓ represents lower values in COVID-19 patients compared to controls; ↑ represents higher values in COVID-19 patients compared to controls; ARF—acute respiratory failure; ≈ represents no statistically significant differences. Empty box means that no measurement was performed.

**Table 2:** Modulation of RAS molecules in COVID-19 patients during hospitalization, stratified based on the severity of the disease.

COVID-19		ACE (Act.)	ACE (Conc.)	ACE2 (Act.)	ACE2 (Conc.)	AngII (Conc.)	Ang1-7 (Conc.)	AngII/Ang1-7 (Ratio)	Ref.
Severe, *n* = 16	Non-severe, *n* = 120	↓							[60]
Severe, *n* = 59	Non-severe, *n* = 128			↑					[68]
Severe, *n* = 32	Non-severe, *n* = 94					↑			[69]
Severe, *n* = 109	Non-severe, *n* = 196				↑				[70]
Severe/critical, *n* = 40	Mild, *n* = 42					↑			[63]
Severe, *n* = 11	Mild, *n* = 24	≈							[57]
Severe, *n* = 263	Mild, *n* = 82				↓				[71]
Severe, *n* = 11	Critical, *n* = 12		≈		≈	↑	≈		[66]

Act.—activity; Conc.—concentration; Ref.—reference; ↓ represents lower values in COVID-19 patients compared to controls; ↑ represents higher values in COVID-19 patients compared to controls; ≈ represents no statistically significant differences. Empty box means that no measurement was performed.

**Table 3:** Modulation of RAS molecules in COVID-19 patients during hospitalization, stratified based on the outcome of the disease.

COVID-19		ACE (Act.)	ACE (Conc.)	ACE2 (Act.)	ACE2 (Conc.)	AngII (Conc.)	Ang1-7 (Conc.)	AngII/Ang1-7 (Ratio)	Ref.
Died, *n* = 25	Survived, *n* = 49			≈	≈	≈	≈	↑	[50]
Died, *n* = 11	Survived, *n* = 17				↑				[74]
ICU/died, *n* = 260	Transferred/discharged, *n* = 331				↓				[52]
Mechanical ventilation/died, *n* = 106	Non-ventilated survivors, *n* = 136				≈				[55]
ICU, *n* = 8	Discharched, *n* = 22					≈			[56]
Intubated, *n* = 79	Non-intubated, *n* = 225				↑				[70]

Act.—activity; Conc.—concentration; Ref.—reference; ↓ represents lower values in COVID-19 patients compared to controls; ↑ represents higher values in COVID-19 patients compared to controls; ≈ represents no statistically significant differences. Empty box means that no measurement was performed.

## Data Availability

The datasets generated and analyzed during the current study are available in the Supplementary Materials.

## Supplementary Materials

The following supporting information can be downloaded at: https://www.mdpi.com/article/10.3390/microorganisms12030583/s1, Supplementary Data Set; Supplemental-S1: MATERIAL AND METHODS: Bibliography research strategy, Study population [75,100], ACE2 Activity [101], ACE Activity, Angiotensin II and Angiotensin 1-7, List of reagent, Statistical analyses; Table S1: Demographic characteristics of the study population; Table S2: Results of univariable logistic regression models; Table S3: Multivariable logistic regression analysis to identify variables associated with ACE, ACE2, Ang 1-7, and AngII variability among COVID-19 patients. Only variables significant (*p* < 0.2) in the univariable analysis were included in the multivariable model.
