# Supplementary material for: The Renin–Angiotensin System (RAS) in COVID-19 Disease: Where We Are 3 Years after the Beginning of the Pandemic
Source: Microorganisms. 2024 Mar 14;12(3):583. doi: 10.3390/microorganisms12030583 (PMC10975343; doi:10.3390/microorganisms12030583)
Supplement: Supplementary file 1 [file microorganisms-12-00583-s001.zip › microorganisms-2873710-supplementary.pdf]

Review

The renin-angiotensin system (RAS) in COVID-19 disease: where we are 3 years after the beginning of the pandemic

Marco Prato[1], Natalia Tiberti[1], Cristina Mazzi[2], Federico Gobbi[1], Chiara Piubelli[1] and Silvia Stefania Longoni[1]*

1 Department of Infectious, Tropical Diseases and Microbiology, IRCCS Sacro Cuore Don Calabria Hospital, Negrar di Valpolicella, Verona, Italy
2 Centre for Clinical Research, IRCCS Sacro Cuore - Don Calabria Hospital, Negrar di Valpolicella, Verona, Italy
* Correspondence: silvia.longoni@sacrocuore.it

MATERIAL AND METHODS

Bibliography research strategy

The reference for this review were retrived from PubMed up to December 2023, the research was undertook selecting articles found using as entry terms: SARS, MERS, viral infection, SARS-CoV2, COVID-19, RAS, ACE, ACE2, AngII, Ang1-7, serum, plasma, systemic, circulating.
Specifically: “circulating RAS” or “ circulating renien angiotensin system”,“circulating ACE” or “circulating Angiotensin Converting Enzyme”, “cirucalting ACE2” or “ circulating Angiotensin Converting Enzyme 2”, “circulating AngII” or “circulating Angiotensin II”, “circulating Ang1-7” or “circulating Angiotensin 1-7”, “systemic RAS” or “ systemic renien angiotensin system”, “systemic ACE” or “systemic Angiotensin Converting Enzyme”, “systemic ACE2” or “systemic Angiotensin Converting Enzyme 2”, “systemic AngII” or “systemic Angiotensin II”, “systemic Ang1-7” or “systemic Angiotensin 1-7”, “serum RAS” or “serum renien angiotensin system”, “serum ACE” or “serum Angiotensin Converting Enzyme”, “serum ACE2” or “serum Angiotensin Converting Enzyme 2”, “serum AngII” or “serum Angiotensin II”, “serum Ang1-7” or “serum Angiotensin 1-7”, “plasma RAS” or “plasma renien angiotensin system”, “plasma ACE” or “ plasma Angiotensin Converting Enzyme”, “plasma ACE2” or “plasma Angiotensin Converting Enzyme 2”, “plasma AngII” or “plasma Angiotensin II”, “plasma Ang1-7” or “plasma Angiotensin 1-7”.

Study population

Serum samples (n=47) from COVID-19 patients admitted to the IRCCS Sacro Cuore Don Calabria Hospital in Negrar (Verona, Italy) during the first COVID-19 wave in the period from March to April 2020 were investigated. Patients were stratified into three categories of severity based on a modified WHO score (WHO Working Group on the Clinical Characterisation and Management of COVID-19 infection, 2020), as already reported elsewhere [1]. Briefly, patients were classified as: mild (score 4, n=18), moderate (score 5, n=23), severe (score≥6, n=6). Patients were aslo stratified based on the clinical course during hospitalization (i.e, worsened (n=19) or improved (n=28)). Demographic, clinical characteristics and laboratory findings are described in Table S1 and Supplementary Data Set. Sars-CoV-2 RT-PCR, used for COVID-19 diagnosis, was performed applying the CDC 2019-nCoV rRT-PCR Diagnostic Panel assay and protocol (available at https://www.fda.gov/media/134922/download) [2]. Blood specimens were collected upon admission

and serum stored at -80°C until their use. Twelve serum samples from non-COVID-19 volunteers (6 female and 6 male), median age 54 (interquartile range (IQR) of 50-57), were also included.

ACE2 Activity

ACE2 activity was measured by an in-house method [3]. A serial diluted (1:2 in Assay Buffer) [50mM MES, 300mM NaCl, 10µM $ZnCl_2$] standard curve [range 50-1.56ng/ml], using human recombinant ACE2 (rACE2) (Sigma-Aldrich, Merck KGaA, Darmstadt, Germany), was tested in duplicate. Serum sample, at final volume of 5µl, was analysed in duplicate. Standard curve and serum samples were run simultaneously and in duplicate, in the presence of 10^{-5}M ACE2 Inhibitor (MLN-4760). The assay was performed by adding to each well 75µl of working solution [15µM Mca-Ala-Pro-Lys(Dnp)-OH, 1mM NEM, 1mM PMSF in Assay Buffer], 15µl of standard or serum sample (diluted 1/3) and, 10µl of distilled H_2O or 10^{-5}M ACE2 Inhibitor (MLN-4760). The plate was incubated at room temperature, in the dark, under gently shaking for 16h. The plate was read at 320/405nm (Em/Ex) on a microplate reader (Varioskan LUX, ThermoFisher, Waltham, MA, USA). The concentration of ACE2 was calculated through the equation generated by the standard curve, after subtracting the relative fluorescence unit (RFU) of each inhibited standard and sample, as described [3].

ACE Activity

ACE activity was measured using the Angiotensin I Converting Enzyme (ACE) Activity Assay Kit (Fluorimetric) (Sigma-Aldrich, Merck KGaA, Darmstadt, Germany) following the manufacturer's instruction. Briefly, the standard curve [range 0-0.8nmol] was settled to a final volume of 100µl, in duplicate. A final volume of 5µl of serum samples was tested in duplicate. To each unknown samples, 45µl of assay buffer and 50µl of substrate were added. Serum samples were assessed in duplicate, in the presence of 50µM ACE inhibitor (Captopril). ACE kinetic activity was read at 320/405nm (Em/Ex) on a Varioskan LUX microplate reader at intervals of 60 seconds for 5 minutes. The mU of ACE was calculated through an Excel sheet provided by the manufacturer, after RFU subtraction of each inhibited standards and samples.

Angiotensin II and Angiotensin 1-7

Angiotensin II and Angiotensin 1-7 were measured using the specific ELISA kits Human Angiotensin II (AngII) ELISA Kit (MybioSource, Inc., San Diego, CA, USA) with a detection range of 31.25-2000pg/mL, and Human Angiotensin 1-7 ELISA Kit (MybioSource, Inc., San Diego, CA, USA) with detection range of 15.6pg/ml-500pg/ml, following the instruction of the manufacturer. Standards and serum samples were analysed in duplicate and on a Varioskan LUX microplate reader at 450nm. The Optical Densities (OD) was used to back-calculate the concentration of each molecules based on the standard curve, as per manufacturer's instructions.

List of reagent

MES (Sigma-Aldrich M3671),
NaCl (Sigma-Aldrich 55886),
$ZnCl_2$ (Sigma-Aldrich 746355),
Mca-Ala-Pro-Lys(Dnp)-OH (Bachem AG, 4042638),
NEM (Sigma-Aldrich 04259),
PMSF (Sigma-Roche PMFS-RO),
rACE2 (Sigma-Aldrich SAE0064),
MLN-4760 (Sigma-Aldrich 3464873),

Captopril (Sigma-Aldrich C4042)
Black-96-well PP plate (Sigma-Aldrich M9685)
Angiotensin I Converting Enzyme (ACE) Activity Assay Kit (Fluorometric) (Sigma-Aldrich CS0002),
Human Angiotensin II (AngII) ELISA Kit (MybioSource Inc., MBS2506893)
Human Angiotensin 1-7 ELISA Kit (MybioSource Inc., MBS084052)

Statistical analyses

All collected data were summarized using descriptive statistics. Estimated parameters are reported with 95% confidence interval (CI), categorical data are presented as count and percentages while continuous data as median with interquartile range. The assumption of normality was tested using Shapiro-Wilk test. The statistical significance, fixed at 5%, was estimated applying Man-Whitney or Kruskal-Wallis tests. Both statistical methods and plots were used to assess test results. Correlation between continuous variables was tested using Spearman's correlation coefficient. Box and Cox transformation was performed in case of non-normal dependant variables. Simple and multiple linear regression were applied to describe the relationship between ACE and ACE2 enzymes and AngII and Ang1-7 peptides with covariates of interest (i.e. age, gender, disease severity, presence of comorbidity and therapy with ACE inhibitors or angiotensin receptor blockers). Significance threshold for the univariate linear regression was set at p-value 0.2. Analyses and plots were performed with SAS EG v7.1 (SAS Institute Inc., NC, USA) and GraphPad Prism v8.3.0 (GraphPad Software, CA, USA).

Table S1. Demographic characteristics of the study population

	**COVID-19 (n=47)**	**NON-COVID-19 (n=12)**
**Age, Median (IQR)**	75 (68-76)	53.5 (52-57.25)
**Gender M, n (%)**	26 (55%)	6 (50%)
**ACEi/ARB therapy, Yes, n (%)**	14 (30%)	n/a
**Clinical course, Improved n (%)**	28 (60%)	n/a
**Clinical outcome, Discharged n (%)**	39 (83%)	n/a
**Severity, n (%)**[a]		n/a
Mild	18 (38%)	
Moderate	23 (49%)	
Severe	6 (13%)	
**Co-morbidities, n (%)**	38 (81%)	n/a
Diabetes	14 (30%)	
Cardiovascular diseases	21 (46%)	
Hypertension	19 (41%)	
Neoplasm	4 (9%)	
Respiratory diseases	9 (20%)	
Neurodegenerative diseases	2 (4%)	
Hormonal and metabolic disorder	4 (9%)	

Obesity	3 (7%)	
Other chronic diseases	6 (13%)	
**Fever (yes), n (%)**	21 (45%)	n/a
**Diarrhea (yes), n (%)**	4 (8.5%)	n/a
**Cough (yes), n (%)**	12 (25.5%)	n/a
**PaO_2/FiO_2 (mmHg), median [IQR]**	275 (211.5-329)	n/a
**Oxygen supply yes, n (%)**	37 (79%)	n/a
**Type of oxygen supply, n (%)**		n/a
Low flow	35 (75%)	
NIV	1 (2%)	
Intubation	1 (2%)	
**Heart rate (beats/minute), median [IQR]**	79 (70 – 86)	n/a
**Systolic_bp (mmHg), median [IQR]**	120 (110 – 133.75)	n/a
**Dyastolic_bp (mmHg), median [IQR]**	70 (65 – 80)	n/a
**Ongoing treatment (yes), n (%)**[b]	33 (70%)	n/a
**Laboratory parameters at admission**[c]		n/a
LDH, U/L	272 (202 - 335)	
VES, mm/H*	76.5 (53 - 92.25)	
IL6, pg/mL	44.67 (25.36 - 87.02)	
CRP, mg/L	76.54 (19.45 - 133.3)	
D dimer, µg/L*	988.5 (497.3 - 2221.0)	
Creatinine, mg/dL	0.85 (0.74 - 1.22)	
WBC, 10^9/L	5.7 (4.2 - 7.8)	
Neutrophils, 10^9/L*	4.4 (2.475 - 5.575)	
Lymphocytes, 10^9/L*	1 (0.7 - 1.5)	

IQR = Interquartile Range; **ACEi/ARB** = ACE inhibitors/ angiotensin receptor blockers; [a] Mild: modified WHO score=4; Moderate: modified WHO score=5; Severe: modified WHO score≥6; **PaO_2** = Arterial Blood Oxygen tension; **FiO_2**= Fraction of Inspired Oxygen; **NIV** = Non-Invasive Ventilation; [b] Full list of the specific treatment for each patients are available in supplementary Data Set; [c] Parameters retrieved from each patients database: **LDH** = Lactate Dehydrogenase, **VES** = Velocity Erythrocytes Sedimentation, **IL6** = Interleukin 6, **CRP** = C-reactive Protein, **WBC** = White Blood Cells; * Missing data for 1 patient; n/a = not available

Table S2. Results of univariable logistic regression models.

		ACE (mU)		ACE2 (mU)		Ang1-7 (pg/ml)		AngII (pg/ml)	
**Characteristic**	**N**	**Beta [95% CI]**	**p-value**	**Beta [95% CI]**	**p-value**	**Beta [95% CI]**	**p-value**	**Beta [95% CI]**	**p-value**
**ACE inhibitors**	47		0.804		**0.002**		**0.006**		0.662
No		—		—		—		—	
Yes		0.15 [-1.0, 1.3]		0.91 [0.36, 1.5]		-12 [-20, -3.7]		0.02 [-0.06, 0.09]	
**Age**	47	0.00 [-0.04, 0.04]	0.931	0.02 [0.00, 0.03]	**0.101**	-0.21 [-0.49, 0.07]	**0.132**	0.00 [0.00, 0.00]	0.627
**Gender**	47		**0.048**		**0.051**		0.964		**0.022**
M		—		—		—		—	
F		1.1 [0.01, 2.1]		-0.54 [-1.1, 0.00]		0.19 [-8.1, 8.5]		0.07 [0.01, 0.14]	
**Disease severity**	47		0.228		0.204		0.338		0.317
Mild		—		—		—		—	
Moderate or severe		-0.65 [-1.7, 0.42]		0.36 [-0.20, 0.91]		3.9 [-4.2, 12]		-0.03 [-0.10, 0.03]	
**Comorbidity**	47		0.780		**0.013**		**0.094**		**0.009**
No		—		—		—		—	
Yes		0.19 [-1.2, 1.6]		0.86 [0.19, 1.5]		-8.6 [-19, 1.5]		-0.11 [-0.18, -0.03]	

CI = Confidence Interval; ACE: Angiotensin-converting enzyme. In bold are reported variables with a p-value<0.2

Table S3. Multivariable logistic regression analysis to identify variables associated with ACE, ACE2, Ang 1-7 and AngII variability among COVID-19 patients. Only variables significant (p<0.2) in the univariable analysis were included in the multivariable model.

		ACE2 (mU)		Ang1-7 (pg/ml)		AngII (pg/ml)	
**Characteristic**	**N**	**Beta [95% CI]**	**p-value**	**Beta [95% CI]**	**p-value**	**Beta [95% CI]**	**p-value**
**ACE inhibitors**	47		**0.012**		**0.027**		
No		—		—			
Yes		0.75 [0.17, 1.3]		-10 [-20, -1.2]			
**Age**	47	0.00 [-0.02, 0.02]	0.741	-0.04 [-0.35, 0.28]	0.815		
**Gender**	47		0.075				0.060
M		—				—	
F		-0.47 [-1.0, 0.05]				0.06 [0.00, 0.12]	
**Comorbidity**	47		0.292		0.460		**0.024**
No		—		—		—	
Yes		0.39 [-0.35, 1.1]		-4.1 [-15, 7.1]		-0.09 [-0.17, -0.01]	
**R^2 adjusted**		0.24		0.12		0.17	

CI = Confidence Interval; ACE: Angiotensin-converting enzyme.

References

1. Caldrer, S.; Mazzi, C.; Bernardi, M.; Prato, M.; Ronzoni, N.; Rodari, P.; Angheben, A.; Piubelli, C.; Tiberti, N., Regulatory T Cells as Predictors of Clinical Course in Hospitalised COVID-19 Patients. *Frontiers in immunology* **2021,** *12*, 789735.
2. Marcolungo, L.; Beltrami, C.; Degli Esposti, C.; Lopatriello, G.; Piubelli, C.; Mori, A.; Pomari, E.; Deiana, M.; Scarso, S.; Bisoffi, Z.; Grosso, V.; Cosentino, E.; Maestri, S.; Lavezzari, D.; Iadarola, B.; Paterno, M.; Segala, E.; Giovannone, B.; Gallinaro, M.; Rossato, M.; Delledonne, M., ACoRE: Accurate SARS-CoV-2 genome reconstruction for the characterization of intra-host and inter-host viral diversity in clinical samples and for the evaluation of re-infections. *Genomics* **2021,** *113* (4), 1628-1638.
3. Xiao, F.; Burns, K. D., Measurement of Angiotensin Converting Enzyme 2 Activity in Biological Fluid (ACE2). *Methods in molecular biology* **2017,** *1527*, 101-115.
